# Effect of Bovine Tuberculosis on Selected Productivity Parameters and Trading in Dairy Cattle Kept Under Intensive Husbandry in Central Ethiopia

**DOI:** 10.3389/fvets.2021.698768

**Published:** 2021-07-21

**Authors:** Rea Tschopp, Andrew J. K. Conlan, Gizachew Gemechu, Gizat Almaw, Jan Hattendorf, Jakob Zinsstag, the ETHICOBOTS consortium, James L. N. Wood

**Affiliations:** ^1^Department of Epidemiology and Public Health, Swiss Tropical and Public Health Institute, Basel, Switzerland; ^2^University of Basel, Basel, Switzerland; ^3^One-Health Unit, Armauer Hansen Research Institute, Addis Ababa, Ethiopia; ^4^Disease Dynamics Unit, Department of Veterinary Medicine, University of Cambridge, Cambridge, United Kingdom; ^5^National Animal Health Diagnostic and Investigation Center, Sebeta, Ethiopia

**Keywords:** Ethiopia, dairy cattle, productivity, bovine tuberculosis, disease impact

## Abstract

Bovine tuberculosis (BTB) has substantial impact on fertility, milk, and meat productivity in cattle. However, these assumptions are based on outdated data. Recent global studies on the impact of BTB on cattle productivity are scarce and show sometimes inconclusive and/or contradicting results. This pilot study is the first longitudinal study performed in urban upgraded dairy cattle in Ethiopia that are kept under intensive husbandry. We assessed whether BTB has an impact on various animal productivity parameters and animal movement. Animals (*N* = 890) included in the study were tested for BTB at least once using the comparative intradermal tuberculin test (CIDT). Fertility, mortality, and offtake were assessed in 21 dairy farms where herd follow-ups over 3 years were performed. In addition, an independent abattoir survey was conducted to assess carcass weight and visible TB-like lesions upon meat inspection. Animal movements (purchasing and offtakes) were documented for each farm. The impact of BTB status on the intervals been birth, service, and calving times and the intercalving intervals was analyzed using a Cox proportional hazards model. The hazard ratio associated with BTB-positive animals was smaller than 1 for all fertility parameters, suggesting that BTB status increases the time between events; however, the effect was small and only statistically significant (95% level) for the time between calving and service. Offtakes included a higher percentage of reactor animals (58%) as compared with non-reactor animals (42%) (*p* = 0.0001). Overall, reactors were eliminated from the farms within 238.6 days after receiving test results, which was 54.9 days faster than for negative animals. The majority of owners purchased animals within their town or its surrounding. Nearly a quarter of reactors were sold directly to other farms. Animals were also sold further away, including other regions, raising the question of disease spread and the need for an animal tracing mechanism. In the abattoir survey, a total of 349 carcasses were weighed, of which 8% showed visible TB-like lesions and 53.6% had fasciolosis. Negative adult bull carcasses were 7.5 kg heavier than reactor bulls.

## Introduction

Bovine tuberculosis (BTB) is a chronic bacterial zoonotic disease caused by *Mycobacterium bovis*, with cattle being the primary host. However, a wide-range of domesticated and wild mammal species as well as humans can become infected with BTB through direct contact with a positive host or consumption of raw animal products (1, 2).

Besides being an animal and public health concern, BTB has globally substantial direct and indirect economic implications related to direct impacts on animal health and productivity, surveillance-control and eradication programs, market losses, and animal movement restrictions.

Costs of BTB control and interventions have been quantified in some contexts (3–6). The economic cost of BTB on animal level and productivity, on the other hand, has rarely been captured. The literature mentions that BTB causes increased mortality, reduced milk and meat productivity, reduced fertility, and organ or carcass condemnations at abattoirs due to visible TB-like lesions. However, references of quantifications are sparse. The most extensive analysis on the BTB impact on animal productivity was carried out by Meisinger (7), who followed up 8,000 cows over 5 years and conducted an abattoir survey in former East Germany in the 1970s. He found an average milk loss of 10% and a meat production loss of between 6 and 12%. Bernues et al. (8) assumed a milk loss of 12% and a reduction in fertility of 5%. The Meisinger and Bernues figures were since then extensively used to describe productivity and economic losses in cattle due to BTB. However, these figures are now outdated and do not take into account other factors such as breed, husbandry, and environment.

More recent small-scale studies showed diverging or sometimes inconclusive results. Boland et al. (9) showed a significant decrease in milk production in BTB reactors in Irish dairy farms ranging from 120 to 573 kg milk loss per lactation. Rahman and Samad (10) showed a 17% decrease in milk yield in Bangladesh, but their study included only 17 lactating cows. On the other hand, more recently, a study done in Mexico (11) showed only marginal decrease of some reproductive performances and milk yields in BTB-positive animals. Organ and carcass condemnation due to TB-like visible lesions has a cost implication at abattoir level (12–15). However, studies focused generally on TB visible lesions during meat inspection and rarely followed up on laboratory diagnostics to confirm *M. bovis*.

Cattle productivity analyses have rarely been performed. Such analysis require large animal numbers, longitudinal studies, or very well kept detailed records on productivity parameters over years. Studies in the scale of the Meisinger study have never been replicated in recent times. In Africa, only very rare longitudinal studies were performed to estimate fertility and mortality of local zebu cattle under traditional husbandry management (16–19).

BTB is endemic in Ethiopia (20, 21). Overall, low prevalence was described in local zebu cattle (2, 22). In contrast, BTB has been shown to be an important disease in exotic breeds and their crosses in Ethiopia particularly when kept under intensive husbandry systems. In and around Addis Ababa, BTB prevalence in dairy cattle ranged between 11.4 and 53.6% (23–26). BTB prevalence in emerging intensive dairy areas in the country ranged between 1.4 and 12% (27). Few studies exist in Africa at large and in Ethiopia in particular on BTB impacts on animal productivity. A small retrospective record-based study investigated the impact on calving rates (28).

This present study is the first longitudinal study performed in urban dairy cattle in Ethiopia that are being kept under intensive husbandry. We aimed in this pilot study to assess whether BTB has an impact on animal movement and on various animal productivity parameters and, through establishing biological effect sizes, inform the necessary sampling scale for future larger studies.

## Materials and Methods

The methods used are similar to the ones described in Tschopp et al. (29) and are therefore summarized here for brevity.

### Study Sites

This study was carried out in Central Ethiopia within the greater milk production belt, namely, Addis Ababa (Kaliti, Kolfe, Yeka, and Gulele districts), as well as Debre Zeit and Sendafa, 50 and 40 km away from the capital, respectively. Data were collected between November 2015 and September 2018. The area is conducive to dairy farming and has high production potential. It benefits from a temperate climate and an abundant rainfall (1,000–1,900 mm/year) with good animal fodder potential and holds the largest high-yielding dairy cow numbers in the country. The milk shed of the study areas has access to big markets including Addis Ababa (30).

### Study Farms and Animals

Intensive urban dairy farms were selected based on the willingness of the owners to collaborate on a 3-year longitudinal study. Animals were high milk-yielding Holstein–Frisian cross-breed cattle. All farm sizes were represented and categorized into small farms (three to 19 animals), medium farms (20–49 animals), and large farms (50 and more animals). Except for one government farm, all farms were privately owned. Husbandry was similar within farm size categories.

Animals were categorized into sex and age class. Young stock were animals younger than 12 months, replacement stock were animals from 12 months to around 3 years, and breeders were from 3 years onwards.

### Tools and Parameters Recorded

An initial registration of all animals was performed in each farm at the start of the study. A herd-book was prepared capturing parameters such as any new animal entry (purchase, birth, and gift), animal exit (selling, slaughtering, and death), detailed data related to selling and purchasing of animals (e.g., cost, location, and reason), morbidities including mastitis, mortalities, and fertility [artificial insemination (AI) dates and calving dates]. The farms were visited twice a month by the same investigators. Herd-book information was updated on hard copies during each visit, and data were entered in a Microsoft Access table. All animals were dewormed once a year with albendazole.

Animals were tested early in the study (spring 2016) using the comparative intradermal tuberculin test (CIDT). They were tested again toward the end of the study (2018) by a trained veterinarian. Two sites of 12–15 cm apart in the middle of the neck were shaved and cleaned. Skinfold thickness was measured with a digital caliper at both sites. Animals were then injected intradermally with 0.1 ml (2,500 IU/ml) avian PPD and 0.1 ml (3,000 IU/ml) bovine PPD (Lelystad B.V., Overijssel, the Netherlands) using insulin syringes. The injections sites were examined, and the skin thicknesses measured 72 h post-injection. The difference in the increase of skin thickness at the bovine and avian sites before and after inoculation was measured and recorded by the same veterinarian. A reaction was considered positive if the increase in skin thickness at the bovine site of injection was 4 mm greater than at the reaction shown at the site of the avian injection. The reaction was inconclusive if the increase was between 1 and 4 mm (31) and negative if the increase was <1 mm.

### Abattoir Study

An abattoir study was carried out independently from the above longitudinal study (different animal group as above). Carcasses from cattle slaughtered in two abattoirs in the study area (Addis Ababa and Sululta) were weighed by quarters. Liver was examined for *Fasciola hepatica* infection and graded as + low infection, ++ moderate infection, and +++ heavy infection, leading to organ confiscation. Carcasses were also assessed for visible TB lesions, and samples from these lesions were collected and cultured in duplicate on glycerol and 0.4% pyruvate LJ medium (Loewenstein-Jensen TB Medium Base, 500G; BD Cat No. 283813; Sigma-Aldrich Corp., St. Louis, MO, USA) and incubated at 37°C. Cultures were observed daily for growing colonies during the first week and then weekly from the second week onwards. Cultures with no growth at week 8 were considered negative.

### Data Analysis

Raw log book data from farms within the study were entered in Microsoft Access tables. Each fertility parameter can be considered as a time interval, measuring the time to either calving or service from a given reference time (birth or previous calving date). As such, each of these measures is potentially right censored either through the removal of the animal from the herd before the end of the study or by the end of the study itself. Survival analyses specifically account for this censoring and are therefore the most appropriate way to assess the impact of BTB status, or other explanatory factors, on each of these parameters (32). To this end, extracts from the study database were imported into R (33) and reshaped to create tables of time intervals for each fertility parameter (34). For censored observations, the censoring date was taken to either the date of removal of the animal from the herd or the end of the study taken as the date of the final BTB test on December 14, 2018. All R code and data tables are provided as Supplementary Material.

We performed an initial exploratory analysis by plotting the Kaplan–Meier survival curves for each fertility measure in turn, stratified by farm size, farm identification, and TB test status using the survival package in R (35). These revealed larger variations in the hazard rate for each fertility parameter with respect to farm size and between farms than with BTB status, suggesting that these must be adjusted for in assessing the impact of BTB status. We therefore fit a Cox proportional hazards model for each parameter, with BTB status and farm size (categorical) as explanatory variables. Robust errors were calculated using an infinitesimal jackknife variance estimate to adjust for clustering of animals within herds (32, 35). The proportional hazards assumption was assessed by graphical inspection of the scaled Schoenfeld residuals.

As the Cox model only provides the hazard ratio with respect to BTB status, we also present summary statistics for each fertility parameter in terms of the median value and 95% quantiles (which are of intrinsic interest with respect to understanding the baseline productivity within commercial dairy herds in Ethiopia).

### Ethical Clearance

This research study was approved by the Institutional Review Board (IRB) of Aklilu Lemma Institute of Pathobiology, Addis Ababa University (reference number IRB/ALIPB/2018) and the IRB of AHRI (AAERC) (reference number PO46/14) and supported by the Ethiopian Ministry of Livestock and Fisheries. Skin testing of cattle was based on the international standards (31). Verbal informed consent was given by farmer owners, following the disclosure of the project objectives, benefits, and possible limitations. Each owner was informed about the results of their animals on the day when skin reactions were measured.

## Results

### Bovine Tuberculosis Prevalence

A total of 890 animals were tested at least once for BTB in 21 farms. Sixteen farms (76.2%) had at least one reactor animal. Overall, animal crude apparent prevalence for BTB was 40% (*N* = 356) and 31.7% after adjusting by herd size (95% CI: 19.5–47%). Overall, 53.2% (*N* = 474) were negative and 6.8% (*N* = 60) were inconclusive. Three-hundred twenty-seven animals were tested twice over a period of 3 years, of which 237 (72.5%) kept the same BTB status, whereas 42 animals (12.8%) converted from inconclusive or negative to being reactors. An additional eight animals (2.4%) converted from negative into inconclusive. The remaining 40 animals that were initially positive either became inconclusive or negative.

### Bovine Tuberculosis Impact on Reproductive Parameters

The following reproductive parameters were analyzed in regard to BTB status: time between animal birth to the first service, time between animal birth and their first calving, calving interval, and time interval between calving and next first service. Farm size effects were all non-significant (and not reported here) but were forced into the model to adjust for this important source of variation identified by the exploratory analysis. The hazard ratio associated with BTB-positive animals was smaller than 1 for all fertility parameters, suggesting that BTB status increases the time between events. However, the estimated effect was small and only significant (at the 95% level) for the calving to service time (see Table 1). Table 2 shows the median and 95% quantiles for the different fertility parameters stratified by BTB status and farm size.

**Table 1:** Hazard ratios for fertility parameters among BTB-positive cows.

**Parameter**	**Nb of positive animals**	**Nb of negative animals**	**HR**	**95% CI**	***p*-value**
Calving to next service	397	354	0.753	0.58–0.98	0.037
Calving interval	398	354	0.973	0.75–1.26	0.834
Birth to first calving	148	58	0.896	0.28–2.98	0.852
Birth to first service	148	58	0.809	0.47–1.40	0.450

*BTB, bovine tuberculosis*.

**Table 2:** Description of fertility parameters by farm size and BTB status.

**Parameter**	**Farm size**	**BTB status**	***N***	**Median days**	**95% quantiles**
Calving to next AI	Small	Neg	14	126.5	87.6–229.0
		Pos	8	188.0	72.0–295.2
	Medium	Neg	53	139.0	59.5–263.4
		Pos	23	133.0	55.1–269.2
	Large	Neg	124	136.5	27.3–303.4
		Pos	125	133.0	35.3–316.6
	Overall	Neg	191	137.0	34.0–294.5
		Pos	156	133.5	37.6–313.5
Calving interval	Small	Neg	10	435.5	303.6–620.8
		Pos	6	443.5	296.6–824.5
	Medium	Neg	46	431.5	327.7–776.1
		Pos	16	481.5	313.4–687.2
	Large	Neg	85	462.3	306.2–939.9
		Pos	108	473.5	319.4–907.5
	Overall	Neg	141	445.0	303.0–876.0
		Pos	130	474.0	301.1–889.8
Birth to first calving	Small	Neg	3	789.0	705.4–954.3
		Pos	18	–	–
	Medium	Neg	10	690.5	629.7–900.2
		Pos	5	766.0	635.5–1033.0
	Large	Neg	13	818.0	474.6–1066.6
		Pos	6	945.5	799.2–1156.0
	Overall	Neg	26	777.0	488.2–1034.7
		Pos	11	808.0	646.7–1151.0
Birth to first AI	Small	Neg	7	485.0	428.3–660.7
		Pos	26	–	–
	Medium	Neg	19	503.0	381.0–796.3
		Pos	5	511.0	416.8–590.0
	Large	Neg	30	618.0	435.7–847.7
		Pos	19	706.0	485.0–1083.7
	Overall	Neg	56	548.0	390.1–858.7
		Pos	24	666.0	420.6–1060.6

*BTB, bovine tuberculosis; AI, artificial insemination*.

### Bovine Tuberculosis and Natural Mortality

Among the BTB tested animals, 34 animals died of a natural cause during the study period. Seventeen were reactors and 17 were BTB negative. The numbers were too small to justify further analyses.

### Bovine Tuberculosis and Offtakes

Overall, at the end of the study, 245 animals among the 830 tested animals had been eliminated (selling or slaughtering); among them, 103 (42%) were BTB negative and 142 (58%) were BTB positive. Among all 356 BTB-positive animals, 142 were eliminated (39.9%) and 214 (60.1%) were still in the farms at the end of the study. Among the 474 BTB-negative animals, 103 (21.7%) were eliminated and 371 (78.3%) were kept.

Animals tested during the first round were followed up over time to assess whether owners would eliminate them and, if so, how long after the test results. Among the offtakes (*N* = 236), 96 (40.7%) were BTB negative and 140 (59.3%) BTB positive. Overall, for the 236 animals that were eliminated, median time between test result and elimination of the animal was 191.5 days (95% CI: 158–254) regardless of TB status. Overall, reactors were eliminated faster than BTB-negative animals (mean difference was 54.9 days). This was particularly true in medium farms (*p* = 0.02) (see Table 3).

**Table 3:** Time interval between CIDT result and offtake (*N* = 236) among reactor and non-reactor animals.

**Farm size**	**TB status**	**Nb**	**Mean days test to offtake**	**Days difference**	**SE**	**95% CI**	***p*-value**
Small	Neg	0	–				
	Pos	5	154.4	–	72.5		
Medium	Neg	40	242.9		26.3	189.6–296.1	0.02
	Pos	45	170.2	72.7	17.9	134.2–206.2	
Large	Neg	56	329.7		24.6	280.3–379.1	0.14
	Pos	90	277.5	52.2	22.8	232.1–322.9	
Overall	Neg	96	293.5		18.5	256.7–330.3	0.03
	Pos	140	238.6	54.9	16.5	206.0–271.3	

*CIDT, comparative intradermal tuberculin test*.

Detailed information on where these positive animals went was available for 82 animals. The great majority was sent to abattoirs (*N* = 60; 73.2%) mainly in Addis Ababa, whereas a quarter were sold directly to other farms (*N* = 21, 25.6%), and one animal was sold to a broker.

### Bovine Tuberculosis Impact on Carcass Weight

The abattoir study included 351 animals, of which 349 carcasses were weighed. One hundred forty (40.1%) and 209 (59.9%) carcasses were from Addis Ababa and Sululta abattoir, respectively. One hundred ten were male (41.2%) and 157 female (58.8). Replacements accounted for 48 animals (13.7%) and breeders for 299 animals (85.7%).

Overall *Fasciola* prevalence was 53.6% (*N* = 187). Half of the animals had moderate *Fasciola* infestation (*N* = 95; 50.8%), whereas 32.1% (*N* = 60) had severe and 17.1% (*N* = 32) had mild infestation. Twenty-eight (8%) animals had visible TB-like lesions upon meat inspection examination. Culture was done on 18 lesions, and 13/18 (72.2%) were culture positive for *Mycobacteria*.

BTB-negative breeding bull carcasses were overall 7.5 kg heavier than BTB-positive animals; however, there was no statistical difference at the 95% level (see Table 4).

**Table 4:** TB status and carcass weight among 267 animals (based on visible TB-like lesions).

**Animal category**	**TB status**	**Observations**	**Mean weight (kg)**	**Weight difference**	**SE for mean**	**CI for mean**	***p*-value**
Heifer	Pos	1	112.0		–	–	
	Neg	6	96.3	−15.7	17.4	51.6–141.0	
Young bull	Pos	1	123.0		–	–	
	Neg	26	135.5	+12.5	7.9	119.2–151–7	
Breeding cows	Pos	15	127.1		11.2	103.0–151.2	
	Neg	135	126.8	−0.3	2.6	121.6–131.9	0.96
Breeding bulls	Pos	9	143.0		17.6	102.4–183.6	
	Neg	74	155.5	+7.5	5.3	144.9–166.0	0.44

### Animal Trade

Overall, 102 animals were purchased. Among them, 53 were included in the original stock during registration, and 49 were further purchased during the study time for 48 of which detailed information was collected. The majority of animals were purchased directly from another farm (93.7%), while 6.2% were purchased from local markets. No brokers were involved. The majority of farmers bought their animals within their town or its surrounding. Addis Ababa farmers purchased 89.6% of the total purchased animals in the study.

Overall, 538 animals were sold during the study period. The majority were heifers and cows (*N* = 275; 51.1% of all animals sold) followed by male calves that were eliminated within the first 2 weeks of life (*N* = 222, 41.3% of all sold animals). The majority of sold animals originated in the Addis farms (*N* = 299; 55.6% of all sold animals) and the least in Sendafa (*N* = 110; 20.4%). Reasons for selling were given for 467 animals, as follows: immediate need for cash (18.6%), underperforming animals (14.6%), depopulation (10.3%), diseased animals (6.8%), old animals (6%), and animals suffering a trauma such as broken legs (2.4%). The rest were young male calves eliminated within their first 2 weeks of life. TB was never given as a direct reason. Most animals were sold directly to abattoirs (*N* = 264/375, 70.4%). Nearly a quarter (*N* = 90/375, 24%) were sold directly to another farms, whereas 15 animals (4%) were sold *via* brokers. None were sold *via* a market system; 66.6% of all animals were sold to Addis Ababa, where large abattoirs are found. Table 5 shows the locations where animals have been purchased from and/or sold to. The majority of the trade—particularly purchase of animals—remained local within the study site and its neighboring locations. Animals were sold in addition to other regions.

**Table 5:** Purchase and selling locations of animals observed in the three study sites.

**Locations of selling or purchase**		**Selling by study site**				**Purchasing by study site**			
		**Addis Ababa**	**Bishoftu**	**Sendafa**	**Total**	**Addis Ababa**	**Bishoftu**	**Sendafa**	**Total**
Addis Ababa		121 (75.7)	33 (37.9)	57 (81.4)	211 (66.6)	35 (67.3)		1 (20)	36 (62)
Addis Ababa surroundings	Mukatori					8 (15.4)			8 (13.8)
	Lemma					6 (11.5)			6 (10.3)
Bishoftu			20 (23)	2 (2.8)	22 (6.9)		1 (100)		1 (1.7)
Bishoftu surroundings	Adama	32 (20)			7 (2.2)				
Sendafa			2 (2.3)	7 (10)	9 (2.8)			3 (60)	3 (5.2)
Sendafa surroundings	Girar							1 (20)	1 (1.7)
	Bake			3 (4.2)	3 (0.9)				
Debre Berhan (Amhara)				1 (1.4)	33 (10.4)				
Dessie (Amhara)			16 (18.4)		16 (5)				
Mekele (Tigray)			10 (11.5)		10 (3.1)				
Wellega (Oromia)			6 (6.9)		6 (1.9)				
Gondar (Amhara)						3 (5.8)			3 (5.2)
Total		160	87	70	317	52	1	5	58

## Discussion

Overall, crude BTB prevalence was 40% at animal level, which is in line with previous studies performed in the area (23, 24). During the 3-year study time, 12.8% of initially negative animals converted into new reactors. An additional eight animals (2.45%) converted from negative to doubtful, possibly being in the process of becoming positive. In recent times, an increase in number of intensive dairy farms has been observed in several regions of the country (emerging dairy areas). Central Ethiopia remains often the source of animals for stocking up new farms or replacing animals, hence raising the question of possible risk for BTB spread into new areas, particularly in a national context where no BTB control measures exist (e.g., no compulsory testing and no movement restrictions). Our study showed that animal trading (purchasing and selling) was mostly occurring locally within the same town or within the same geographical area. The majority of sold animals were sold to local abattoirs (70.4%). However, nearly a quarter (24%) of animals were sold to other farms mainly in the surroundings but some—albeit few in numbers—also further away, even to other regions of the country (e.g., Tigray and Amhara), hence increasing the risk for further TB spread. Animal trade was observed between dairy farms belonging to universities (from Addis to Mekele and Gondar). Interestingly, none of the reactors included in our study were traded to the various universities. University dairy farms in the different regions would have the strong potential to become further animal stocking/re-stocking source for dairy farms in the respective regions and to act as a model farm that could provide awareness and good farm practice training to existing and emerging dairy farms.

Without institutional and legal support in BTB surveillance and control, further spread of the diseases could potentially be linked to farmers' attitude upon receiving test results. Overall, 39.9% of the reactors were eliminated as compared with 21.7% of the non-reactors. Our study showed that among all 356 BTB reactors, 60.1% were kept in the farms and 39.9% were eliminated during the study time. Regardless of BTB status, the median time interval between a test result and elimination date was 191.5 days (95% CI: 158–254). Overall, reactors were eliminated faster than non-reactors (mean 54.9 days quicker; *p* = 0.03). This result highlights potential substantial hidden economic impact of BTB on farms that cannot be easily measured. There is an indication that farmers are selling more reactors than non-reactors and that they are selling their reactors faster than they would in average. Therefore, they might lose on optimal market prices, affecting both farm dynamic and business through, for instance, economic and logistic challenge of animal sourcing for restocking; loss of markets; and a possible additional factor (among many others) of observed farm depopulation. In our study, nine out of the 24 study farm (37.5%) had their animal number decrease over the last 3 years including 80% of all large farms (29). This study showed also that nearly a quarter of the reactors were sold to other farms and not slaughtered, hence highlighting the risk of disease spread through uncontrolled animal movement.

Limited studies exist on BTB effect on various productivity parameters. BTB has negative impacts on animal fertility (7, 8); however, recent studies on quantifications are lacking. Interestingly, BTB impact on animal reproduction has been analyzed for wildlife bovine species such as the Wood bison in Canada (*Bison bison athabascae)* and the African buffalo (*Syncerus caffer*), where lower pregnancy probability was described (36, 37). But data from domestic cattle are lacking. A preliminary study in Ethiopia based on a retrospective record study showed that BTB status had no influence on age at puberty and age at first calving, but reactor cows in their third calving had significantly higher mean number of services (28). Our results showed that the hazard ratio associated with BTB-positive animals was smaller than 1 for all fertility parameters, suggesting that BTB status increases the time between events. The median interval was overall longer in BTB-positive animals as compared with negative animals (with the exception for the calving to next service parameter).

**Figure 1:**
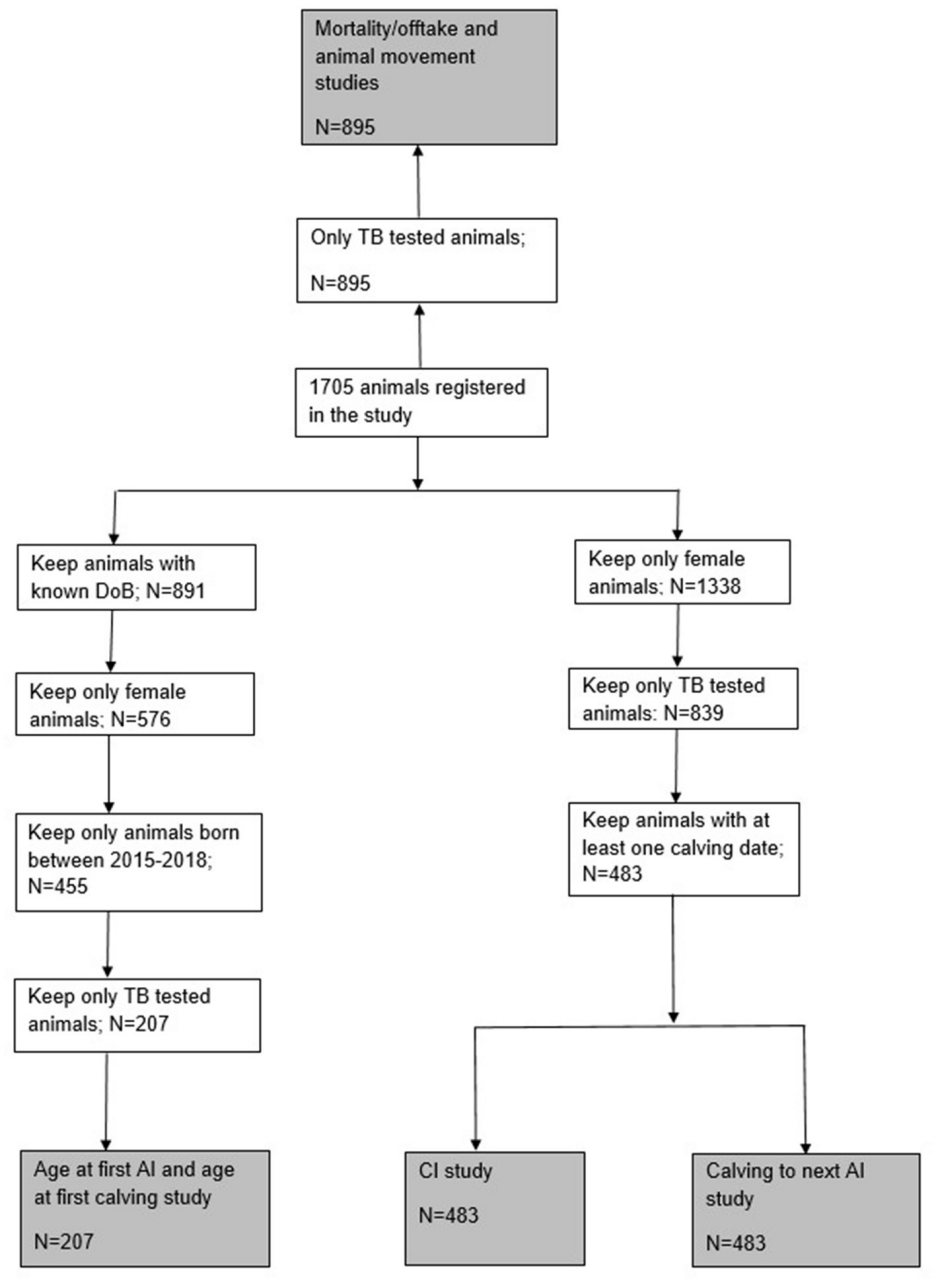
Flowchart showing animals used for each study analysis.

Our study did not take in account specific age of the animal and related lactation number when calculating the fertility parameters. A significant proportion of animals within our study population were lost due to the absence of a TB test result (Figure 1) due to either being too young at the date of the first test or having been removed from the herd by the second. If there is an association between BTB infection status and removal of animals, then this may introduce biases that are difficult to quantify. This is an important, but unavoidable, limitation of carrying out our study under production conditions on commercial farms given the frequency of testing that was feasible (given the nature of the tuberculin test) and acceptable to the farmers.

Studies of the economic BTB impact on meat production have so far primarily focused on abattoir study with monetary translation of confiscation of carcasses and organs due to visible TB-like lesions (14, 15, 38–40). BTB (solely based on visible lesions) is one of the major reasons for meat and organ confiscation at abattoirs in Ethiopia besides parasitic diseases (12, 41, 42). However, the direct loss of meat (reduced life weight and reduced carcass weight) has to our knowledge never been assessed in Africa. Unfortunately, our sample size was small (N = 349), with 8% visible TB-like lesions upon meat inspection. Nevertheless, we could observe a trend that negative bulls produced in average 7.5 kg more meat than positive bulls.

Our study showed also that half of the animals (53.6%) were infested with *Fasciola* (*F. hepatica*), a common liver fluke found in cattle. A third (32.1%) showed even severe infestation of the liver. *F. hepatica* infections were shown to be linked with altered immune responsiveness to PPD, hence having implications with BTB diagnosis and disease progression (43–45). The high prevalence of *Fasciola* observed will need to be addressed before embarking in large-scale future BTB surveillance and control programs in Ethiopia. In a nation with no compulsory TB testing, a mechanism that can trace back carcasses from abattoirs with visible TB-lesions to the source farms would be an additional important step in TB surveillance.

## Conclusion

This study is the first one in Ethiopia to assess the potential impact on BTB on dairy cattle productivity parameters in a holistic way (fertility, mortality, morbidity, and weight). Due to the lack of accurate record keeping of milk yield, we could not calculate the effect of BTB on milk productivity. Although monetary translation of productivity loss was difficult to perform at this stage, and although our sample size was too small for definitive conclusions, our pilot study indicated that BTB has likely an impact on productivity (lower fertility and weight loss) and has likely further invisible costs and indirect economic impacts such as forgoing access to better markets, time, and change in farm business. Besides a regular BTB testing follow-up of each animal over time, a much larger sample size including >5,000 animals is warranted in future follow-up studies to corroborate these findings and enable to control other factors influencing productivity such as husbandry factors or comorbidities. With the growing intensification of cattle production in Ethiopia, analysis of productivity and impact of diseases in productivity are important. The results of this study will provide important information to conduct further cost–benefit analysis of different control strategies for BTB.

## Data Availability Statement

The datasets presented in this article are not readily available because restrictions linked to project (EThicobots) regulations.

## Ethics Statement

This research study was approved by the Institutional Review Board (IRB) of Aklilu Lemma Institute of Pathobiology, Addis Ababa University (reference number IRB/ALIPB/2018), the Institutional Review Board of AHRI (AAERC) (reference number PO46/14) and supported by the Ethiopian Ministry of Livestock and Fisheries. Written informed consent for participation was not obtained from the owners because Verbal informed consent was obtained by owners.

## Author Contributions

RT designed the research, performed the data analysis, and drafted the manuscript. GG and GA collected field data and contributed to drafting the manuscript. AC and JH contributed to the data analysis. JW and JZ provided critical comments to the manuscript. All authors have contributed to the manuscript and have approved the submitted version.

## The ETHICOBOTS Consortium

The members of the Ethiopia Control of Bovine Tuberculosis Strategies (ETHICOBOTS) consortium are as follows: Abraham Aseffa, Adane Mihret, Bamlak Tessema, Bizuneh Belachew, Eshcolewyene Fekadu, Fantanesh Melese, Gizachew Gemechu, Hawult Taye, Rea Tschopp, Shewit Haile, Sosina Ayalew, and Tsegaye Hailu, all from Armauer Hansen Research Institute, Ethiopia; Rea Tschopp from Swiss Tropical and Public Health Institute, Switzerland; Adam Bekele, Chilot Yirga, Mulualem Ambaw, Tadele Mamo, and Tesfaye Solomon, all from Ethiopian Institute of Agricultural Research, Ethiopia; Tilaye Teklewold from Amhara Regional Agricultural Research Institute, Ethiopia; Solomon Gebre, Getachew Gari, Mesfin Sahle, Abde Aliy, Abebe Olani, Asegedech Sirak, Gizat Almaw, Getnet Mekonnen, Mekdes Tamiru, and Sintayehu Guta, all from National Animal Health Diagnostic and Investigation Center, Ethiopia; James Wood, Andrew Conlan, and Alan Clarke, all from Cambridge University, United Kingdom; Henrietta L. Moore and Catherine Hodge, both from University College London, United Kingdom; Constance Smith at the University of Manchester, United Kingdom; R. Glyn Hewinson, Stefan Berg, Martin Vordermeier, and Javier Nunez-Garcia, all from Animal and Plant Health Agency, United Kingdom; Gobena Ameni, Berecha Bayissa, Aboma Zewude, Adane Worku, Lemma Terfassa, Mahlet Chanyalew, Temesgen Mohammed, and Miserach Zeleke, all from Addis Ababa University, Ethiopia.

## Conflict of Interest

The authors declare that the research was conducted in the absence of any commercial or financial relationships that could be construed as a potential conflict of interest.

## References

[1] Fitzgerald SD Kaneene JB. Wildlife reservoirs of bovine tuberculosis worldwide: hosts, pathology, surveillance, and control. Vet Pathol. (2012) 50:488–99. 10.1177/0300985812467472 23169912

[2] Gumi B Schelling E Berg S Firdessa R Erenso G Mekonnen W. Zoonotic transmission of tuberculosis between pastoralists and their livestock in south-east Ethiopia. EcoHealth. (2012) 9:139–49. 10.1007/s10393-012-0754-x 22526748 PMC3415617

[3] Butler A Lobley M Winter M. Economic Impact Assessment of Bovine Tuberculosis in the South West of England. CRPR Research Paper No 30. Centre for Rural Policy Research, Department of Politics, University of Exeter (2010). Available online at: www.centres.ex.ac.uk/crpr/publications/

[4] Smith RL Tauer LW Sanderson MW Grohn YT. Minimum cost to control bovine tuberculosis in cow-calf herds. Prev Vet Med. (2014) 115:18–28. 10.1016/j.prevetmed.2014.03.014 24703601 PMC4076834

[5] Caminiti A Pelone F LaTorre G De Giusti M Saulle R Mannocci A. Control and eradication of tuberculosis in cattle: a systematic review of economic evidence. Vet Rec. (2016) 179:70–5. 10.1136/vr.103616 27422918

[6] Verteramo Chiu LJ Tauer LW Smith RL Grohn YT. Assessment of the bovine tuberculosis elimination protocol in the United States. J Dairy Sci. (2018) 102:2384–400. 10.3168/jds.2018-14990 30692003

[7] Meisinger G. Economic effects of the elimination of bovine tuberculosis on the productivity of cattle herds. 2. Effect on meat production. Monatsh. Veterinarmed. (1970) 25:7–13. 5519247

[8] Bernues A Manrique E Maza MT. Economic evaluation of bovine brucellosis and tuberculosis eradication programmes in a mountain area of Spain. Prev Vet Med. (1997) 30:137–49. 10.1016/S0167-5877(96)01103-8 9234417

[9] Boland F Kelly GE Good M More SJ. Bovine tuberculosis and milk production in infected dairy herds in Ireland. Prev Vet Med. (2010) 93:153–61. 10.1016/j.prevetmed.2009.09.021 19896227

[10] Rahman MA Samad A. Prevalence of bovine tuberculosis and its effects on milk production in Red Chittagong cattle. Bangladesh J Vet Med. (2009) 6:175–8. 10.3329/bjvm.v6i2.2332

[11] Mellado M Resendiz D Martinez AM De Santiago MA Veliz FG Garcia JE. Milk yield and reproductive performance of Holstein cows testing positive for bovine tuberculosis. Tropical Animal Health Production. (2015) 47:1061–6. 10.1007/s11250-015-0828-1 25894823

[12] Alembrhan A Haylegebriel T. Major causes of organ condemnation and economic loss in cattle slaughtered at Adigrat municipal abattoir, northern Ethiopia. Vet World. (2013) 6:734–8. 10.14202/vetworld.2013.734-738

[13] Ejeh EF Raji MA Bello M Lawan FA Francis MI Kudi AC. Prevalence and direct economic losses from bovine tuberculosis in Makurdi, Nigeria. Vet Med Int. (2014) 6. 10.1155/2014/904861 24987543 PMC4060539

[14] Jemalo A Haile G Furgasa W. Major causes of organ condemnation and their economic loss in beef cattle slaughtered at assella municipal abattoir. J Vet Sci Anim Husbandry. (2018) 6:208.

[15] Adelakun OD Akinseye VO Adesokan HK Cadmus SIB. Prevalence and economic losses due to bovine tuberculosis in cattle slaughtered at Bodija municipal abattoir, Ibadan, Nigeria. Folia Vet. (2019) 63:41–7. 10.2478/fv-2019-0006

[16] Itty P Zinsstag J Ankers P Njie M Pfister K. Productivity and Profitability of Village Livestock Enterprises in West Africa: Cattle Production in the Gambia (Manuscript). Abidjan: CSRS (1996).

[17] Agyemang K Dwinger RH Little DA Rowlands GJ. Village N'Dama Cattle Production in West Africa: Six Years of Research in the Gambia. Nairobi; Banjul: International Livestock Research Institute; International Trypanotolerance Centre (1997). p. 131.

[18] Zinsstag J Ankers P Dempfle L Njie M Kaufmann J Itty P. Effect of strategic gastrointestinal nematode control on growth of N'Dama cattle in The Gambia. Vet Parasitol. (1997) 68:143–53 10.1016/S0304-4017(96)01024-2 9066060

[19] Tschopp R Aseffa A Zinsstag J. Cattle productivity under traditional village husbandry system in sellale, central Ethiopia: a four and a half year herd follow-up. Int J Agric Innov Res. (2014) 2:2319−473.

[20] Tschopp R Aseffa A. Bovine tuberculosis and other Mycobacteria in animals in Ethiopia: a systematic review. Jacobs Publish. (2016) 2:026.

[21] Sibhat B Asmare K Demissie K Ayelet G Mamo G Ameni G. Bovine tuberculosis in Ethiopia: a systematic review and meta-analysis. Prev Vet Med. (2017) 147:149–57. 10.1016/j.prevetmed.2017.09.006 29254713 PMC5739073

[22] Tschopp R Schelling E Hattendorf J Young D Aseffa A Zinsstag J. Repeated representative cross-sectional skin testing for bovine tuberculosis in cattle in traditional husbandry system in Ethiopia. Vet Rec. (2010) 167:250–6. 10.1136/vr.c3381 20710033

[23] Tsegaye W Aseffa A Mache A Mengistu Y Berg S Ameni G. Conventional and molecular epidemiology of bovine tuberculosis in dairy farms in Addis Ababa city, the capital of Ethiopia. Int J Appl Res Vet Med. (2010) 8:143–51.

[24] Firdessa R Tschopp S Wubete A Sombo M Hailu E Erenso G. High prevalence of bovine tuberculosis in dairy cattle in central Ethiopia: implications for the dairy industry and public health. PLoS ONE. (2012) 7:e52851. 10.1371/journal.pone.0052851 23285202 PMC3532161

[25] Biru A Ameni G Sori T Desissa F Teklu A Tafess K. Epidemiology and public health significance of bovine tuberculosis in and around Sululta District, Central Ethiopia. Afr J Microbiol Res. (2014) 8:2352–8. 10.5897/AJMR2013.6325

[26] Endalew AM Deresa B Ameni G. Bovine tuberculosis prevalence, potential risk factors and its public health implication in selected state dairy farms, central Ethiopia. World's Vet J. (2017) 7:21–9. 10.5455/wvj.20170290

[27] Mekonnen GA Conlan AJK Berg S Ayele BT Alemu A Guta S. Prevalence of Bovine tuberculosis and its associated risk factors in the emerging dairy belts of regional cities in Ethiopia. Prev Vet Med. (2019) 168:81–9. 10.1016/j.prevetmed.2019.04.010 31097127 PMC10364076

[28] Ameni G Bekele S Tolosa T. Preliminary study on the impact of Bovine Tuberculosis on the reproductive efficiency and productivity of Holstein dairy cows in Central Ethiopia. Bull Anim Health Prod Afr. (2010) 58:222–6. 10.4314/bahpa.v58i3.64210

[29] Tschopp R Gemechu G Wood J the ETHICOBOTS Consortium. A longitudinal study of cattle productivity in intensive dairy farms in Central Ethiopia. Frontiers. (in press). 10.3389/fvets.2021.698760 PMC8387621 34458355

[30] Fransesconi GN Ruben R. The hidden impact of cooperative membership on quality management: a case study from the dairy belt of Addis Ababa. J Entrepreneurial Org Divers. (2012) 1:85–103. 10.5947/jeod.2012.005

[31] Office International des Epizooties (OIE). Terrestrial Manual: Bovine Tuberculosis. (2009). Available online at: http://www.oie.int/fileadmin/Home/eng/Health_standards/tahm/3.04.06_BOVINE_TB.pdf

[32] Therneau TM Grambsch PM. Modeling Survival Data: Extending the Cox Model. New York, NY: Springer (2020).

[33] R Core Team. R: A Language and Environment for Statistical Computing. Vienna: R Foundation for Statistical Computing (2020). Available online at: https://www.R-project.org/

[34] Wickham H Averick M Bryan J Chang W D'Agistino McGowan L François R. Welcome to the tidyverse. J Open Source Softw. (2019) 4:1686. 10.21105/joss.01686

[35] Therneau T. (2020) A Package for Survival Analysis in R. R Package Version 3.2-7. Available online at: https://CRAN.R-project.org/package=survival

[36] Joly DO Messier F. The effect of bovine tuberculosis and brucellosis on reproduction and survival of wood bison in Wood Buffalo National Park. J Anim Ecol. (2005) 74:543–51. 10.1111/j.1365-2656.2005.00953.x

[37] Jolles AE Cooper DV Levin SA. Hidden effects of chronic tuberculosis in African buffalo. Ecology. (2005) 86:2358–64. 10.1890/05-0038

[38] Kwaghe AV Ameh AJ Ambali AG Kudi AC Kachalla MG. Prevalence and economic losses from bovine tuberculosis in Maiduguri, Borno State, Nigeria. Int J Life Sci. (2015) 4:283–7.

[39] Elmonir W Ramadan H. Abattoir based prevalence, economic losses and veterinarians' high-risk practices survey of bovine tuberculosis in mid-delta of Egypt. Alexandria J Vet Sci. (2016) 49:24–30. 10.5455/ajvs.224513

[40] Kumbe A. Financial loss caused by organ condemnation in cattle slaughtered at Asella Municipal Abattoir. J Vet Med Res. (2019) 6:1172.

[41] Sheferaw D Abdu K. Major causes of organ and carcass condemnation and associated financial losses in cattle slaughtered at Kombolcha ELFORA abattoir from 2008-2012, Ethiopia. Ethiopian Vet J. (2017) 21:54–66. 10.4314/evj.v21i1.5

[42] Beyene A. Causes of organ condemnation and economic loss of cattle in developing countries. Int J Eng Dev Res. (2017) 5:776−96.

[43] Flynn RJ Mulcahy G Welsh M Cassidy JP Corbett D Milligan C. Co-Infection of cattle with *Fasciola hepatica* and *Mycobacterium bovis*- immunological consequences. Transboundary Emerg Dis. (2009) 56:269–74. 10.1111/j.1865-1682.2009.01075.x 19575746

[44] Claridge J Diggle P McCann CM Mulcahy G Flynn R McNair J. Fasciola hepatica is associated with the failure to detect bovine tuberculosis in dairy cattle. Nat Commun. (2012) 3:853. 10.1038/ncomms1840 22617293 PMC3989536

[45] Munyeme M Munang'andu HM Nambota A Muma JB Phiri AM Nalubamba KS. The nexus between bovine tuberculosis and fasciolosis infections in cattle of the kafue basin ecosystem in Zambia: implications on abattoir surveillance. Vet Med Int. (2012) 6. 10.1155/2012/921869 23213629 PMC3504483

