# Supplementary material for: Effect of Bovine Tuberculosis on Selected Productivity Parameters and Trading in Dairy Cattle Kept Under Intensive Husbandry in Central Ethiopia
Source: Front Vet Sci. 2021 Jul 21;8:698768. doi: 10.3389/fvets.2021.698768 (PMC8334357; doi:10.3389/fvets.2021.698768)
Supplement: Supplementary file 1 [file Data_Sheet_1.zip › Analysis/Analysis.docx]

Survival Analysis of effect of bovine tuberculosis on selected productivity parameters

Andrew J K Conlan

25/05/2021

## Load and reshape data

Starting point for this analysis is three extracts from log book of farms within study: ‘Calving interval.xlsx’, ‘Calving to next AI.xlsx’ and ‘DOB to first calving and first AI.xlsx’. From these extracts we reshape the data to construct four tables for survival analysis of four fertility measures: Calving to service time (calve_to_AI_surv_intervals_df), Calving interval (calving_surv_intervals_df), Birth to first calving (birth_to_calve_df), Birth to first service (birth_to_ai_df).

Note that for the first two measures there are multiple events per individual, while the latter two measure a single event per animal. Each measure is potentially right censored through either removal of the animal from the herd before the end of the study - or the end of the study itself. A survival analysis is therefore the most appropriate way to assess the impact of bTB status. To this end we fit a cox-proportional hazards model with bTB status and Farm Size as explanatory variables, with an infinitesimal jacknife variance estimate to account for clustering of animals within herds (Therneau 2020, Therneau & Grambsch 2000).

As the cox model only provides the hazard ratio with respect to bTB status we also present summary statistics for each fertility parameter in terms of the median value and 95% quantiles (which are of intrinsic interest with respect to understanding the baseline productivity within commercial dairy herds in Ethiopia).

#### Note on coding for categorical variables

Sex: 1=female, 2=male

Age: 1=calf (up to 1 year), 2= replacements, 3=breeders (as of 3 years), 4= old breeders (over 10 years)

Age was recorded when the animal is registered (entry in the farm), either new born or purchased. Over the 3 years of study age categories will change (a calf becomes replacement, replacement become breeder)

Exit type: 1=natural death, 3=slaughtered, 4=sold (offtake=sold and or slaughter)

Farm size: 1=small, 2=medium, 3=large

BTB_tot: TB status coded as negative (0), positive (1) or doubtful (2). TB positivity based on existence of at least one positive TB test.

#### Calving to service time

In total 1,705 animals were registered. In order to calculate this measure, animals must be female (reducing to 1,337), have been tested at least once during the observation period (leaving 839 animals) and have calved at least once leaving a total of 483 animals recorded in ‘calving to next AI.xlsx’.

As each animal may experience multiple events over the duration of the study we reshape the data in terms of time intervals, with time1 corresponding to a calving date and time 2 corresponding to the next recorded service (AI) date. The boolean “event” variable codes whether an event occured (TRUE) or was censored (by either removal of the animal from the herd or the end of the study taken as the date of the final bTB test 2018-12-14). Dates were converted to days measured from the first recored calving time.

## # A tibble: 751 x 8
## id Farm_id Farm_size BTB Exit_date time1 time2 event
## <dbl> <fct> <fct> <lgl> <dttm> <drtn> <drtn> <lgl>
## 1 1698 21 3 TRUE 2018-12-14 00:00:00 3038 days 3212 days FALSE
## 2 1686 21 3 TRUE 2018-12-14 00:00:00 2273 days 2620 days TRUE
## 3 1686 21 3 TRUE 2018-12-14 00:00:00 2973 days 3212 days FALSE
## 4 356 14 2 FALSE 2018-12-14 00:00:00 1921 days 3212 days FALSE
## 5 1677 21 3 TRUE 2018-12-14 00:00:00 2228 days 2330 days TRUE
## 6 1677 21 3 TRUE 2018-12-14 00:00:00 2611 days 3212 days FALSE
## 7 235 10 2 TRUE 2018-12-14 00:00:00 2744 days 3212 days FALSE
## 8 1543 33 1 FALSE 2018-12-14 00:00:00 2318 days 2442 days TRUE
## 9 1543 33 1 FALSE 2018-12-14 00:00:00 2816 days 3212 days FALSE
## 10 341 14 2 FALSE 2018-12-14 00:00:00 1777 days 3212 days FALSE
## # … with 741 more rows

#### Intercalving time

In total 1,705 animals were registered. In order to calculate this measure, animals must be female (reducing to 1,337), have been tested at least once during the observation period (leaving 839 animals) and have calved at least once leaving a total of 483 animals. (Note there are 484 records in ‘calving to next AI.xlsx’, but animal ID=1770 has no calving dates).

As each animal may experience multiple events over the duration of the study we reshape the data in terms of time intervals, with time1 corresponding to a calving date and time 2 corresponding to the next recorded calving date. The boolean “event” variable codes whether an event occured (TRUE) or was censored (by either removal of the animal from the herd or the end of the study taken as the date of the final bTB test 2018-12-14). Dates were converted to days measured from the first recorded calving time.

## # A tibble: 752 x 8
## id Farm_id Farm_size BTB Exit_date time1 time2 event
## <dbl> <fct> <fct> <lgl> <dttm> <drtn> <drtn> <lgl>
## 1 1698 21 3 TRUE 2018-12-14 00:00:00 3038 days 3212 days FALSE
## 2 1686 21 3 TRUE 2018-12-14 00:00:00 2273 days 2973 days TRUE
## 3 1686 21 3 TRUE 2018-12-14 00:00:00 2973 days 3212 days FALSE
## 4 356 14 2 FALSE 2018-12-14 00:00:00 1921 days 3212 days FALSE
## 5 1677 21 3 TRUE 2018-12-14 00:00:00 2228 days 2611 days TRUE
## 6 1677 21 3 TRUE 2018-12-14 00:00:00 2611 days 3212 days FALSE
## 7 235 10 2 TRUE 2018-12-14 00:00:00 2744 days 3212 days FALSE
## 8 1543 33 1 FALSE 2018-12-14 00:00:00 2318 days 2816 days TRUE
## 9 1543 33 1 FALSE 2018-12-14 00:00:00 2816 days 3212 days FALSE
## 10 341 14 2 FALSE 2018-12-14 00:00:00 1777 days 2758 days TRUE
## # … with 742 more rows

#### Birth to service (AI)

In total 1,705 animals were registered, but only 891 animals had exact birth records. In order to calculate this measure animals must be female (reducing to 576) and we require a birth date within one year of the beginning of the study to ensure animals had not calved previously. Removing animals that did not receive a bTB test leaves a data set of 207 animals for analysis (recorded in ‘DOB to first calving and first AI.xlsx’). Finally, Animal with ID 9999 has a first calving date equal to their birth date so were removed from this and following table to give a final number of 206 animals.

As there is only one possible event for each animal for this measure, we reshape the data in terms of the interval between birth (time1) and service (time2) with the “event” variable encoding whether AI occurred within the observation period (TRUE) or was censored (FALSE). Censoring date is either the removal date of the animal from the herd or the date of the end of the study as before.

## # A tibble: 206 x 7
## ID Farm_id Farm_size BTB time1 time2 event
## <chr> <fct> <fct> <lgl> <dttm> <dttm> <lgl>
## 1 747 34 1 FALSE 2015-10-01 00:00:00 2017-01-28 00:00:00 TRUE
## 2 645 30 2 FALSE 2015-06-02 00:00:00 2016-10-17 00:00:00 TRUE
## 3 881 20 1 FALSE 2015-02-24 00:00:00 2018-12-14 00:00:00 FALSE
## 4 84 35 3 FALSE 2015-04-25 00:00:00 2016-10-28 00:00:00 TRUE
## 5 83 35 3 FALSE 2015-04-03 00:00:00 2017-04-02 00:00:00 TRUE
## 6 748 34 1 FALSE 2015-02-23 00:00:00 2016-04-25 00:00:00 TRUE
## 7 635 30 2 FALSE 2015-01-17 00:00:00 2016-02-28 00:00:00 TRUE
## 8 638 30 2 TRUE 2015-03-24 00:00:00 2016-11-11 00:00:00 TRUE
## 9 639 30 2 FALSE 2015-06-05 00:00:00 2016-10-24 00:00:00 TRUE
## 10 641 30 2 TRUE 2015-08-20 00:00:00 2016-10-17 00:00:00 TRUE
## # … with 196 more rows

#### Birth to calving

In total 1,705 animals were registered, but only 891 animals had exact birth records. In order to calculate this measure animals must be female (reducing to 576) and we require a birth date within one year of the beginning of the study to ensure animals had not calved previously. Removing animals that did not receive a bTB test leaves a data set of 207 animals for analysis (recorded in ‘DOB to first calving and first AI.xlsx’).

As there is only one possible event for each animal for this measure, we reshape the data in terms of the interval between birth (time1) and calving (time2) with the “event” variable encoding whether AI occurred within the observation period (TRUE) or was censored (FALSE). Censoring date is either the removal date of the animal from the herd or the date of the end of the study as before.

## # A tibble: 206 x 7
## ID Farm_id Farm_size BTB time1 time2 event
## <chr> <fct> <fct> <lgl> <dttm> <dttm> <lgl>
## 1 747 34 1 FALSE 2015-10-01 00:00:00 2018-12-14 00:00:00 FALSE
## 2 645 30 2 FALSE 2015-06-02 00:00:00 2017-07-17 00:00:00 TRUE
## 3 881 20 1 FALSE 2015-02-24 00:00:00 2017-01-25 00:00:00 TRUE
## 4 84 35 3 FALSE 2015-04-25 00:00:00 2018-04-25 00:00:00 TRUE
## 5 83 35 3 FALSE 2015-04-03 00:00:00 2018-12-14 00:00:00 FALSE
## 6 748 34 1 FALSE 2015-02-23 00:00:00 2017-10-13 00:00:00 TRUE
## 7 635 30 2 FALSE 2015-01-17 00:00:00 2016-11-30 00:00:00 TRUE
## 8 638 30 2 TRUE 2015-03-24 00:00:00 2018-02-18 00:00:00 TRUE
## 9 639 30 2 FALSE 2015-06-05 00:00:00 2017-12-02 00:00:00 TRUE
## 10 641 30 2 TRUE 2015-08-20 00:00:00 2017-09-30 00:00:00 TRUE
## # … with 196 more rows

## Exploratory analysis of survival data for each fertility measure

To explore the structure of the data we plot Kaplan-Meier survival curves for each fertility measure in turn, stratified by farm size, farm_id and TB test status. These reveal larger variations in the hazard rate for each fertility measure with respect to farm size and between farms than with bTB status.

#### Calving to service time


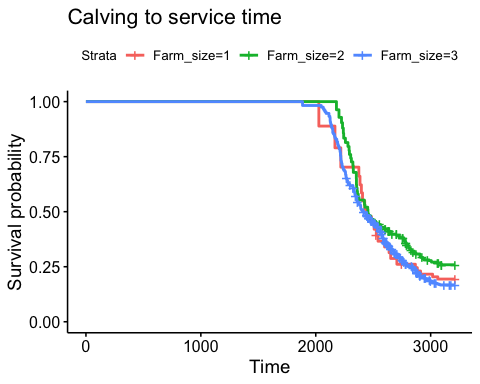

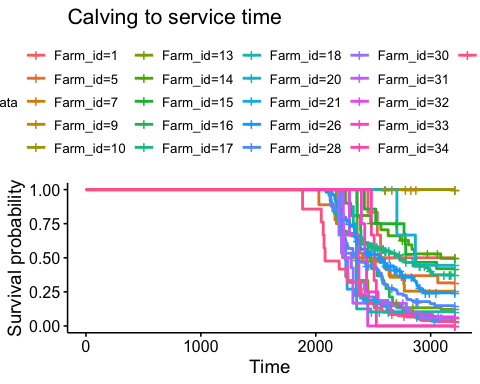

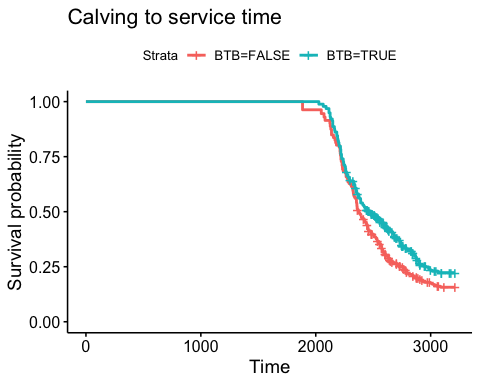


#### Calving interval


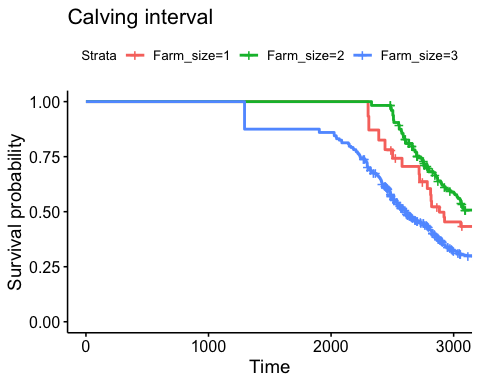

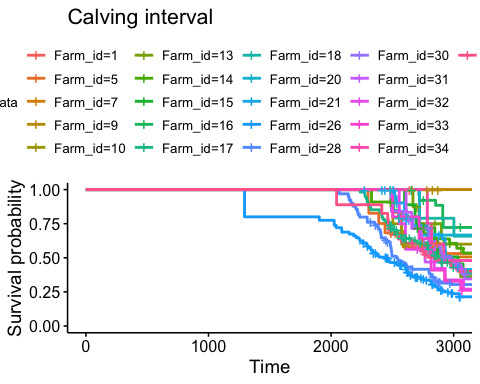

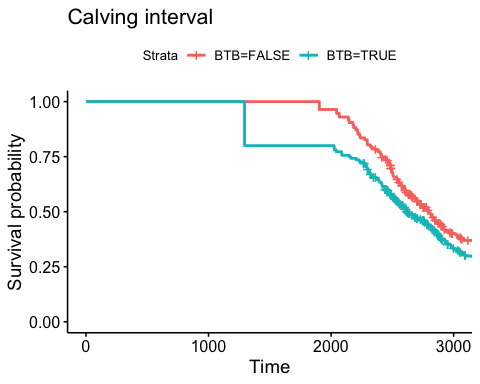


#### Birth to first calving


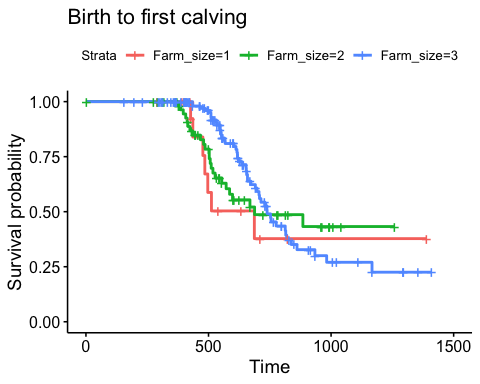

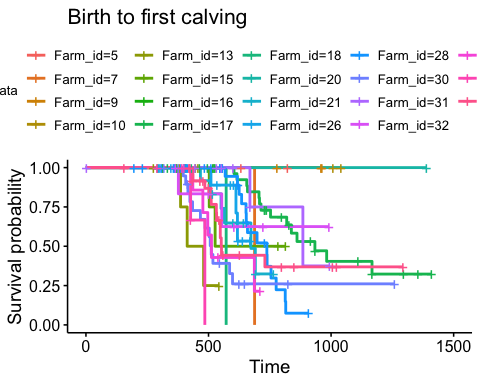

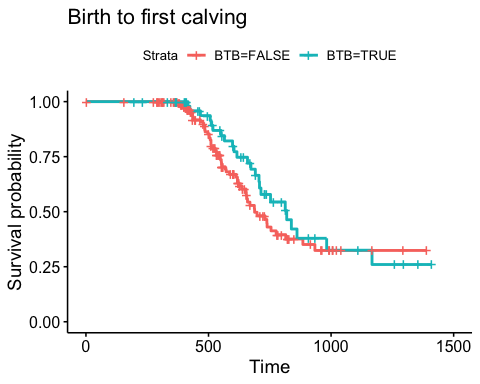


#### Birth to first service


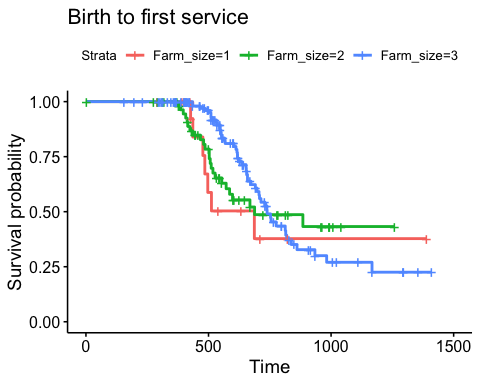

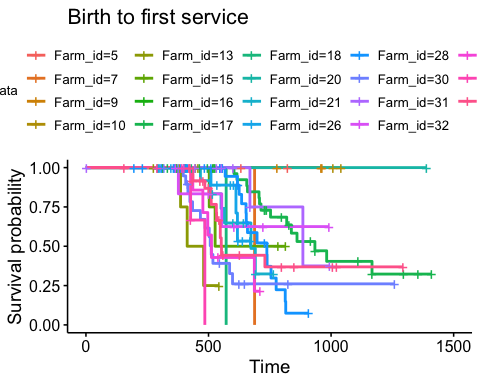

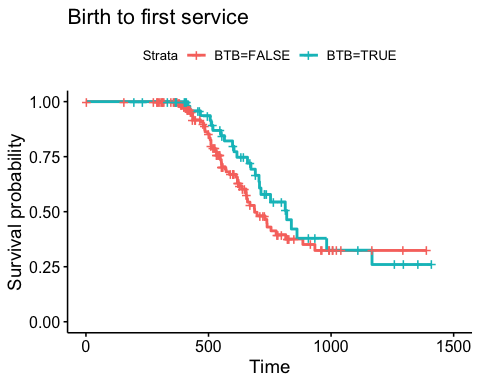
 ## Survival modelling

Exploratory analysis suggests that farm size and between herd variation should be adjusted for in assessing the impact of bTB status. We fit a cox proportional hazards model for each fertility parameter, with bTB status and farm size (categorical) as explanatory variables. Robust errors were calculated using an infinitesimal jacknife variance estimate to adjust for clustering of animals within herds (Therneau 2020, Therneau & Grambsch 2000). The proportional hazards assumption was assessed by graphical inspection of the scaled Schoenfeld residuals.

Farm size effects were all non-significant (and not reported here), but were forced into the model to adjust for this important source of variation. The hazard ratio associated with bTB positive animals is less than 1 for all fertility parameters, suggesting that bTB status increases the time between events. However, the estimated effect is small and only significant (at the 95% level) for the calving to service time.

## # A tibble: 32 x 7
## Parameter Farm_Size BTB_status N Interval Interval_l Interval_h
## <chr> <chr> <chr> <dbl> <dbl> <dbl> <dbl>
## 1 "Calving to servic… "Small" neg 14 126. 87.6 229.
## 2 " " " " pos 8 188 72.1 295.
## 3 " " "Medium" neg 53 139 59.5 263.
## 4 " " " " pos 23 133 55.1 269.
## 5 " " "Large" neg 124 136. 27.3 303.
## 6 " " " " pos 125 133 35.3 317.
## 7 " " "Overall" neg 191 137 34 294.
## 8 " " " " pos 156 134. 37.6 314.
## 9 "Calving interval" "Small" neg 10 436. 304. 621.
## 10 " " " " pos 6 444. 297. 824.
## # … with 22 more rows

## # A tibble: 4 x 7
## Parameter N_pos N_neg HR HR_l HR_h p_value
## <chr> <dbl> <dbl> <dbl> <dbl> <dbl> <dbl>
## 1 Calving to service time 397 354 0.753 0.580 0.98 0.037
## 2 Calving interval 398 354 0.973 0.75 1.26 0.834
## 3 Birth to first calving 148 58 0.896 0.28 2.83 0.852
## 4 Birth to first service 148 58 0.809 0.47 1.4 0.45

## References

Therneau T (2020). *A Package for Survival Analysis in R*. R package version 3.2-7, <URL: <https://CRAN.R-project.org/package=survival>>.

Terry M. Therneau, Patricia M. Grambsch (2000). *Modeling Survival Data: Extending the Cox Model*. Springer, New York. ISBN 0-387-98784-3.
